# Detectable Duration of Viable SARS-CoV-2, Total and Subgenomic SARS-CoV-2 RNA in Noncritically Ill COVID-19 Patients: a Prospective Cohort Study

**DOI:** 10.1128/spectrum.00503-22

**Published:** 2022-05-23

**Authors:** Angsana Phuphuakrat, Ekawat Pasomsub, Sirawat Srichatrapimuk, Suppachok Kirdlarp, Ampa Suksatu, Chanya Srisaowakarn, Suwimon Manopwisedjaroen, Natali Ludowyke, Priyo Budi Purwono, Thongkoon Priengprom, Artit Wongsa, Ammarin Thakkinstian, Suradej Hongeng, Kumthorn Malathum, Arunee Thitithanyanont, Boonrat Tassaneetrithep

**Affiliations:** a Department of Medicine, Faculty of Medicine Ramathibodi Hospital, Mahidol University, Bangkok, Thailand; b Department of Pathology, Faculty of Medicine Ramathibodi Hospital, Mahidol University, Bangkok, Thailand; c Chakri Naruebodindra Medical Institute, Faculty of Medicine Ramathibodi Hospital, Mahidol University, Samut Prakan, Thailand; d Department of Microbiology, Faculty of Science, Mahidol University, Bangkok, Thailand; e Center of Research Excellence in Immunoregulation, Faculty of Medicine Siriraj Hospital, Mahidol University, Bangkok, Thailand; f Department of Clinical Epidemiology and Biostatistics, Faculty of Medicine Ramathibodi Hospital, Mahidol University, Bangkok, Thailand; g Department of Pediatrics, Faculty of Medicine Ramathibodi Hospital, Mahidol University, Bangkok, Thailand; University of Mississippi Medical Center

**Keywords:** COVID-19, infectivity, SARS-CoV-2, viral shedding, subgenomic RNA

## Abstract

Determination of severe acute respiratory syndrome coronavirus 2 (SARS-CoV-2) infectivity is important in guiding the infection control and differentiating between reinfection and persistent viral RNA. Although viral culture is the gold standard to determine viral infectivity, the method is not practical. We studied the kinetics of SARS-CoV-2 total RNAs and subgenomic RNAs (sgRNAs) and their potential role as surrogate markers of viral infectivity. The kinetics of SARS-CoV-2 sgRNAs compared to those of the culture and total RNA shedding in a prospective cohort of patients diagnosed with coronavirus disease 2019 (COVID-19) were investigated. A total of 260 nasopharyngeal swabs from 36 patients were collected every other day after entering the study until the day of viral total RNA clearance, as measured by reverse transcription PCR (RT-PCR). Time to cessation of viral shedding was in order from shortest to longest: by viral culture, sgRNA RT-PCR, and total RNA RT-PCR. The median time (interquartile range) to negativity of viral culture, subgenomic N transcript, and N gene were 7 (5 to 9), 11 (9 to 16), and 18 (13 to 21) days, respectively (*P* < 0.001). Further analysis identified the receipt of steroid as the factors associated with longer duration of viral infectivity (hazard ratio, 3.28; 95% confidence interval, 1.02 to 10.61; *P* = 0.047). We propose the potential role of the detection of SARS-CoV-2 subgenomic RNA as the surrogate marker of viral infectivity. Patients with negative subgenomic N RNA RT-PCR could be considered for ending isolation.

**IMPORTANCE** Our study, combined with existing evidence, suggests the feasibility of the use of subgenomic RNA RT-PCR as a surrogate marker for SARS-CoV-2 infectivity. The kinetics of SARS-CoV-2 subgenomic RNA should be further investigated in immunocompromised patients.

## INTRODUCTION

Coronavirus disease 2019 (COVID-19), which is caused by severe acute respiratory syndrome coronavirus 2 (SARS-CoV-2), has emerged since December 2019, and the World Health Organization (WHO) declared a pandemic in March 2020 (1). The detection of SARS-CoV-2 in patients’ specimens is a crucial step in COVID-19 diagnosis. The gold standard of the diagnosis of COVID-19 is the detection of at least two SARS-CoV-2 gene targets on respiratory tract specimens by a nucleic acid amplification test (NAAT) (2, 3).

Determination of viral infectivity is another important issue in guiding infection control, in both the hospital and the community. In this regard, SARS-CoV-2 isolation from the clinical specimen is the gold standard. Previous studies reported 12 days after the symptom onset as the longest duration of positive viral culture in immunocompetent COVID-19 patients (4). However, prolonged shedding of viable SARS-CoV-2 in immunocompromised patients has been reported (5). In contrast to a brief period of viral infectivity, viral RNA could be detected up to 3 months after the symptom onset (6), and repositivity following negative test results in COVID-19 cases (7, 8) has been reported. These factors make the interpretation of the positive NAAT difficult in certain situations. Additionally, the interpretation of detection of viral RNA after infection is complicated by reinfection (9).

Although viral culture is the best method to identify viable virus and thus determine infectiousness, SARS-CoV-2 culture needs biosafety level 3 facilities, which are not available in most laboratories. Compared to NAAT, viral culture is less sensitive (10). Also, the conclusion of no isolable virus requires up to 14 days, which is impossible for confirmation before releasing every patient. As the pandemic continues and reinfection is increasingly reported, surrogate markers of viral infectivity are needed to differentiate between SARS-CoV-2 reinfection and repositivity.

SARS-CoV-2 is a positive-sense, single-stranded RNA virus that replicates through the generation of negative-sense RNA intermediates that serve as templates for positive-sense genomic RNA (gRNA) and subgenomic RNAs (sgRNAs) (11, 12). sgRNAs are transcribed in infected cells and are poorly packaged into mature virions; thus, their presence could indicate the presence of active viral replication (13). Many studies demonstrated the correlation between sgRNA detection and isolation of viable virus (14–16), but some studies argued against the association (17–19). We aimed to study the kinetics of SARS-CoV-2 total RNAs and sgRNAs and their potential role as surrogate markers of viral infectivity. Subgenomic spike (sgS) and nucleocapsid (sgN) transcripts were investigated. These two sgRNAs were chosen because the former encodes spike protein, which is indispensable for viral entry to target cells, and the latter is the most abundant sgRNA in infected cells (11, 17).

## RESULTS

### Cohort characteristics.

A total of 36 COVID-19 patients agreed to participate in the study. Patient characteristics are presented in Table 1. Twenty-eight (77.8%) patients were women, and the median (interquartile range; IQR) age was 33.5 (27.7 to 45.6) years. All were immunocompetent. One patient had breast cancer and was receiving hormone therapy following surgery. The median duration of symptoms before hospitalization was 3 (2 to 4) days. Fourteen patients (38.9%) received 2-dose vaccination more than 14 days before the diagnosis of COVID-19. One patient (2.8%) was diagnosed with COVID-19 approximately 8 weeks previously and presented with new onset of cough and diarrhea after recent high-risk contact with confirmed COVID-19 cases. Almost all patients (97.2%) were symptomatic at the time of diagnosis. Three patients (8.3%) denied additional swabs before reverse transcription PCR (RT-PCR) negativity. Ten patients (27.8%) had pneumonia, and three patients (8.3%) received oxygen support during hospitalization. None was intubated. Nineteen patients (52.8%) received favipiravir. Five patients (13.9%) received dexamethasone, and one patient (2.8%) received tocilizumab. All patients recovered.

**TABLE 1:** Clinical characteristics of COVID-19 patients in the cohort

Characteristic	Value (N = 36)
Female (*n* [%])	28 (77.8)
Age (yrs) (median [IQR])	33.5 (27.7–45.6)
Body mass index (kg/m^2^) (median [IQR])	25.0 (21.1–29.1)
Underlying disease (*n* [%])	
Hypertension	5 (13.9)
Hyperlipidemia	4 (11.1)
Diabetes mellitus	3 (8.3)
Malignancy	1 (2.8)
Duration of symptom before hospitalization (days) (median [IQR])	3 (2–4)
Complete 2-dose vaccination (*n* [%])	14 (38.9)
Inactivated vaccine	13 (13.1)
Heterologous inactivated/ChAdOx1 nCoV-19 vaccination	1 (2.8)
Symptom at the diagnosis (*n* [%])	35 (97.2)
Pneumonia (*n* [%])	10 (27.8)
Oxygen support (*n* [%])	3 (8.3)
Treatment (*n* [%])	
Favipiravir	19 (52.8)
Dexamethasone	5 (13.9)
Tocilizumab	1 (2.8)
Viral strain identified	
Wild type	4 (11.1)
Alpha	13 (36.1)
Delta	18 (50.0)

### SARS-CoV-2 culture.

**FIG 1:**
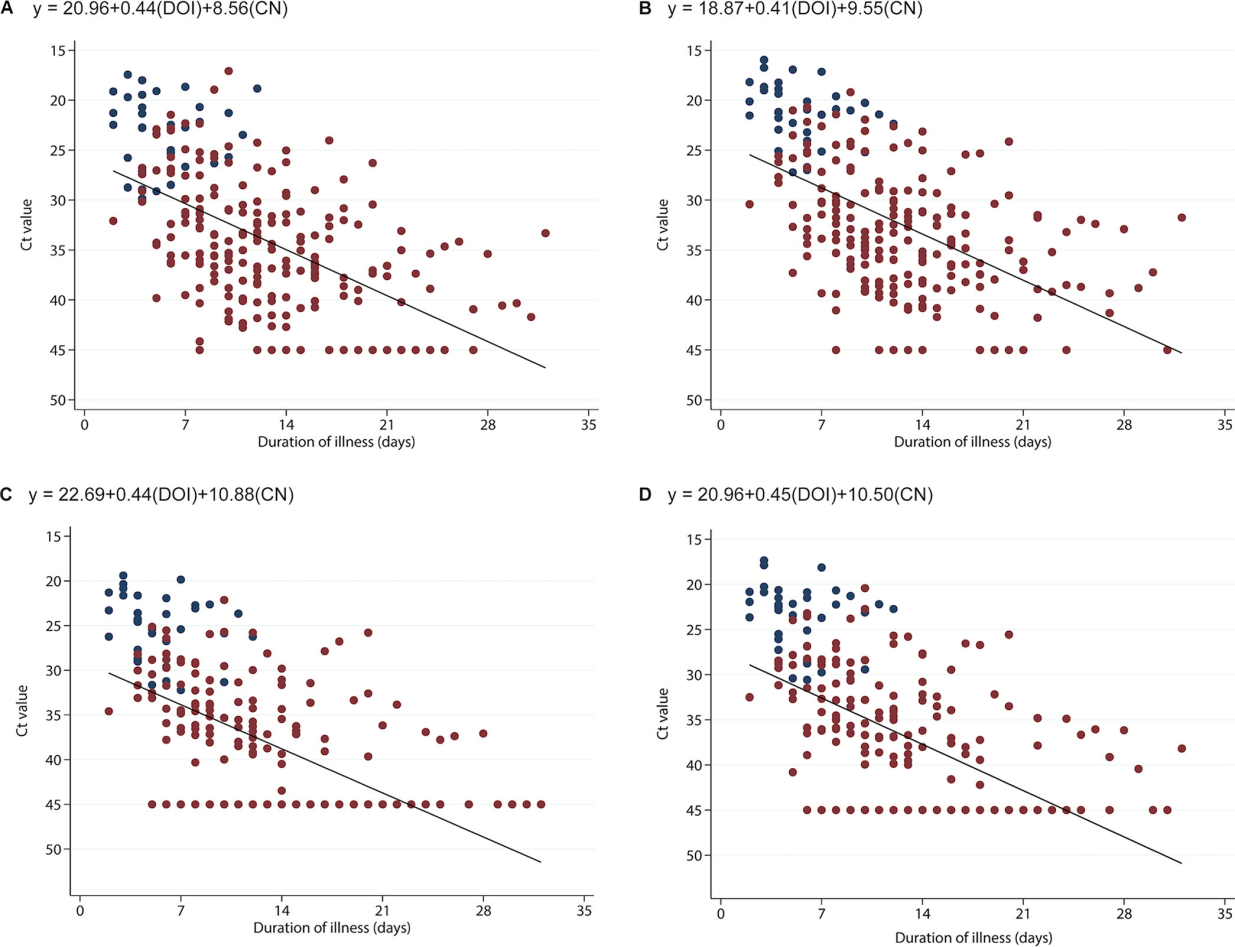
Comparison of cycle threshold (*Ct*) values of ORF1ab (A), total N (B), subgenomic S (C), and subgenomic N (D) versus duration of illness (DOI) in a prospective cohort of COVID-19 patients. Blue and red circles represent samples with isolable and nonisolable viable viruses, respectively. Linear regression equation of each panel is shown in each panel. CN, culture negativity (nonisolable viable virus = 1; isolable viable virus = 0).

A total of 260 nasopharyngeal swab specimens were collected. The median number of swab collection in each patient was 7 (6 to 9) times. Of all specimens collected, 35 specimens (13.5%) yielded positive culture results. Twenty-one patients (58.3%) had positive nasopharyngeal swab culture at least once. In those who had viral culture positivity, the median time of the last positive culture was 6 (4 to 7) days after symptom onset, and the longest duration of positive culture was 12 days after symptom onset (Fig. 1).

Four patients (13.9%) had isolable virus at days 10 to 12 after symptom onset. Of these, three patients had pneumonia; two required supportive oxygen therapy and steroid. Another patient, who had hypertension and dyslipidemia, had only mild upper respiratory tract symptoms.

### SARS-CoV-2 RT-PCR for total RNA and sgRNA.

Cycle threshold (*Ct*) values of RT-PCR for ORF1ab and N genes increased following duration of illness (Fig. 1A and B). The median time to RT-PCR negativity was 18 (14 to 21) days from the date of illness. No virus was recovered in samples with *Ct* values of ORF1ab and N genes higher than 30 and 28, respectively.

*Ct* values of RT-PCR for sgS and sgN also increased following duration of illness (Fig. 1B). The median times to sgS and sgN negativity were 11 (8 to 14) and 11 (9 to 15) days, respectively (Fig. 1C and D). No virus was isolated in samples with *Ct* values of sgS and sgN higher than 33 and 31, respectively.

**FIG 2:**
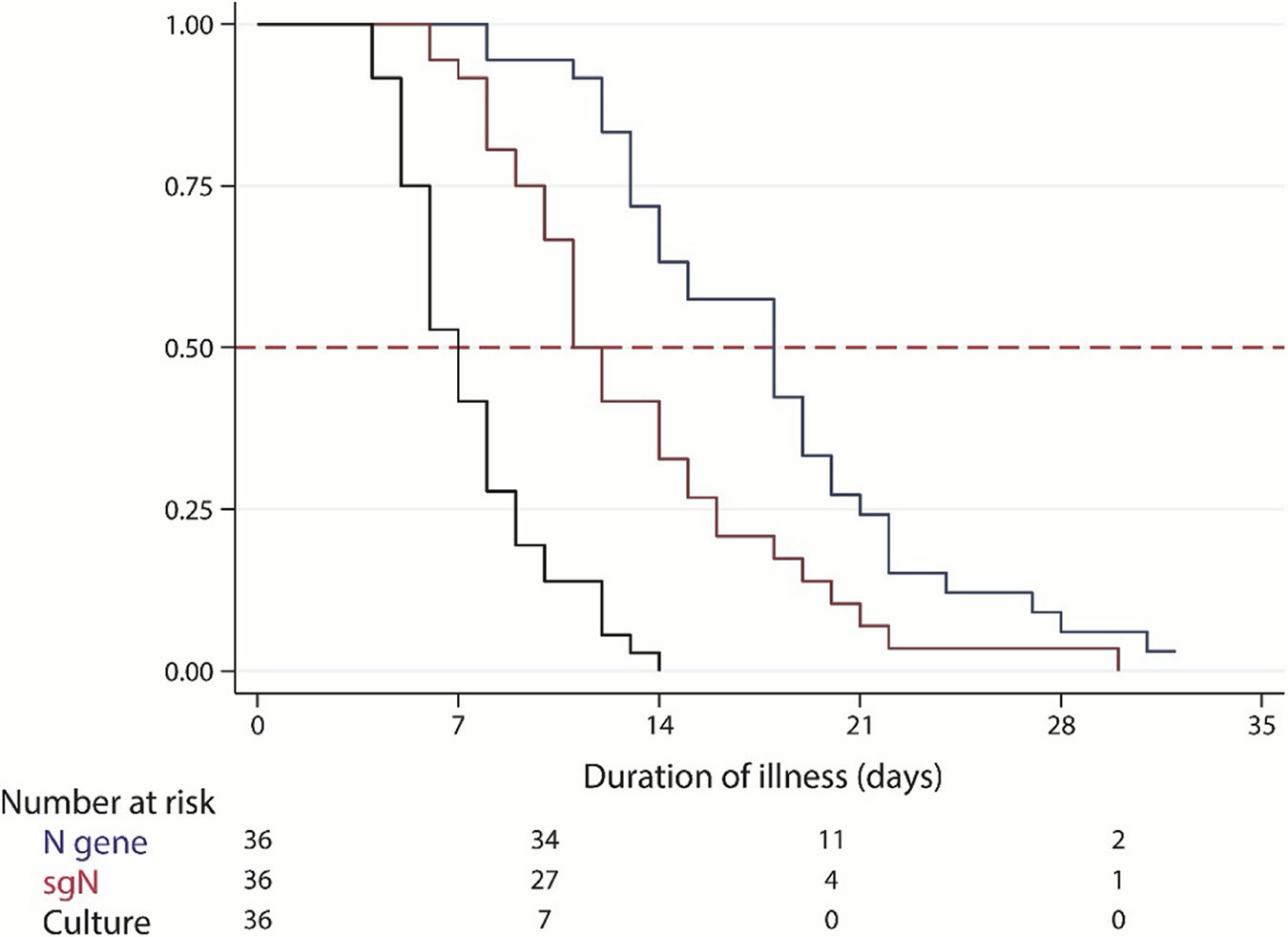
Time to cessation of SARS-CoV-2 viral shedding. Black, blue, and red represent isolable virus, total N RNA, and subgenomic N RNA, respectively.

In order to evaluate the possible use of sgRNA as a surrogate marker for viral infectivity, times to negativity were compared among viral culture, N gene, and sgN. Kaplan-Meier analysis was performed, and log-rank test showed a statistical difference (*P* < 0.001), with culture declined fastest and N gene declined slowest (Fig. 2). sgN declined slower than culture but faster than N gene. The median times to negativity of viral culture, sgN, and N gene were 7 (5 to 9), 11 (9 to 16), and 18 (13 to 21) days, respectively (Fig. 2). Subgroup analysis in cases with isolable virus showed that the median times to negativity of viral culture, sgN, and N gene were 8 (6 to 9), 12 (9 to 18), and 19 (14 to 22) days, respectively.

### Factors associated with duration of viral shedding.

We analyzed factors that might affect duration of isolable virus, and receipt of steroid was associated with time to culture negativity in the univariate cox regression model (hazard ratio [HR], 0.28; 95% confidence interval [CI], 0.09 to 0.83; *P* = 0.022) and remained significant in the multivariate model after adjustment with sex and virus strains (HR, 0.30; 95% CI, 0.09 to 0.98; *P* = 0.047) (Table 2; see Fig. S1A in the supplemental material).

**TABLE 2:** Univariate and multivariate Cox regression analyses of factors associated with time to viral culture negativity

Characteristic	Univariate model			Multivariate model		
	HR	95% CI	*P* value	HR	95% CI	*P* value
Age >40 yrs	0.75	0.38–1.49	0.410			
Female	2.30	0.87–6.13	0.095	1.39	0.48–4.02	0.545
BMI >27.5 (kg/m^2^)	0.97	0.47–1.99	0.934			
Pneumonia	0.60	0.28–1.31	0.202			
Favipiravir	1.17	0.60–2.29	0.652			
Steroid	0.28	0.09–0.83	0.022	0.30	0.09–0.98	0.047
2-dose vaccination	1.48	0.71–3.07	0.291			
Virus strain						
Alpha	0.36	0.11–1.16	0.088	0.47	0.14–1.57	0.219
Delta	0.65	0.22–1.95	0.445	0.81	0.27–2.43	0.707

For the duration of N gene shedding, we found that female sex (HR, 2.98; 95% CI, 1.01 to 8.80; *P* = 0.048), receipt of steroid (HR, 0.21; 95% CI, 0.05 to 0.90; *P* = 0.036), and 2-dose vaccination (HR, 3.08; 95% CI, 1.37 to 6.89; *P* = 0.006) were associated with time to N gene RT-PCR negativity. By multivariate cox regression model, receipt of 2-dose vaccination was the only factor that remained significant (HR, 2.44; 95%, CI 1.10 to 5.43; *P* = 0.028) adjusted by sex (female) and steroid use (Table 3; Fig. S1B).

**TABLE 3:** Univariate and multivariate Cox regression analyses of factors associated with time to RT-PCR negativity of N gene

Characteristic	Univariate model			Multivariate model		
	HR	95% CI	*P* value	HR	95% CI	*P* value
Age >40 yrs	0.60	0.29–1.26	0.177			
Female	2.98	1.01–8.80	0.048	1.80	0.60–5.41	0.296
BMI >27.5 (kg/m^2^)	0.82	0.38–1.77	0.605			
Pneumonia	0.48	0.21–1.12	0.091			
Favipiravir	0.79	0.39–1.59	0.509			
Steroid	0.21	0.05–0.90	0.036	0.34	0.07–1.61	0.175
2-dose vaccination	3.08	1.37–6.89	0.006	2.44	1.10–5.43	0.028
Virus strain						
Alpha	0.67	0.18–2.50	0.549			
Delta	1.83	0.53–6.38	0.341			

For the duration of sgN gene shedding, age of ≥40 years (HR, 0.37; 95% CI, 0.17 to 0.80; *P* = 0.012), female sex (HR, 2.53; 95% CI, 1.01 to 6.38; *P* = 0.049), pneumonia (HR, 0.40; 95% CI, 0.16 to 0.99; *P* = 0.049), and receipt of steroid (HR, 0.19; 95% CI, 0.06 to 0.66; *P* = 0.009) were associated with time to sgN negativity in the univariate model. In the multivariate cox regression model, only receipt of steroid (HR, 0.28; 95% CI, 0.08 to 0.99; *P* = 0.048) remained associated with time to sgN RT-PCR negativity adjusted by age and sex (female) (Table 4; Fig. S1C).

**TABLE 4:** Univariate and multivariate Cox regression analyses of factors associated with time to N gene subgenomic RNA RT-PCR negativity

Characteristic	Univariate model			Multivariate model		
	HR	95% CI	*P* value	HR	95% CI	*P* value
Age >40 yrs	0.37	0.17–0.80	0.012	0.46	0.21–1.01	0.054
Female	2.53	1.01–6.38	0.049	1.82	0.69–4.79	0.224
BMI >27.5 (kg/m^2^)	0.58	0.27–1.26	0.171			
Pneumonia	0.40	0.16–0.99	0.049			
Favipiravir	0.49	0.24–1.01	0.056			
Steroid	0.19	0.06–0.66	0.009	0.28	0.08–0.99	0.048
2-dose vaccination	2.13	0.92–4.93	0.076			
Virus strain						
Alpha	1.15	0.32–4.20	0.830			
Delta	2.19	0.58–8.23	0.245			

## DISCUSSION

Our study in a prospective cohort of predominantly mild COVID-19 patients provided the feasibility of the use of sgRNAs as surrogate markers for SARS-CoV-2 infectivity. We demonstrated the faster decline of sgRNAs compared to total gRNAs.

Understanding the duration of infectiousness and RNA shedding of SARS-CoV-2 is crucial, as this is important for infection prevention policies. At the beginning of the COVID-19 pandemic, test-based strategy for discontinuation of isolation was recommended by WHO, based on the experience with severe acute respiratory syndrome (SARS) and Middle East respiratory syndrome (MERS) (20). For test-based strategy, negative results of RT-PCR from two consecutive specimens were used as a guidance for discontinuing isolation. However, the test-based strategy was, sometimes, confused by persistently or intermittently positive RT-PCR in some patients. Later, the time-based strategy for releasing COVID-19 patients was amended by WHO and U.S. CDC based on the evidence that transmission occurs early in the course of the disease (20, 21). In our cohort, the duration of isolable virus was less than 10 days in all but one mild COVID-19 patient. Although time-based strategy is more practical, the discontinuation of isolation in immunocompromised patients, as well as the interpretation of the detection of viral RNA after disease onset, is challenging.

Viral RNA intermediates, i.e., sgRNAs, have been proposed as a surrogate for viral infectivity (10). Previous studies have investigated the kinetics of subgenomic SARS-CoV-2 RNA during the course of the disease. A study in nine COVID-19 patients with mild illness reported that E gene sgRNA could be detected in throat only within 5 days after symptom onset (22). Another study in 35 patients with mild disease demonstrated that viral E gene sgRNA was rarely detected after 8 days of symptom onset (14). However, one study reported the detection of sgRNAs in 12 SARS-CoV-2-positive oropharyngeal/nasopharyngeal swab samples up to 17 days after initial detection of infection in the next-generation sequencing (NGS) reads (17). This study also demonstrated that sgRNA and virion RNA were stable and were likely protected from nucleases by cellular membranes. In a study with a larger sample size, Verma and coworkers studied N gene sgRNA in 536 samples from 205 patients and reported positivity in 48.1% of participants whose swab was collected 15 to 21 days after symptom onset (23). A subset of positive N gene sgRNA samples was subjected to E gene sgRNA detection; 69.0% of these samples were negative for E gene sgRNA. Another study on E and N sgRNAs from nasopharyngeal swabs obtained from 185 SARS-CoV-2-positive patients revealed the median duration of symptoms to negative test of 14 and 25 days for E and N sgRNAs, respectively (19). The higher abundance of N gene sgRNA compared to that of other sgRNA transcripts and the use of high analytic sensitivity assay, such as NGS, might explain the difference among these studies.

Although total RNA and sgRNA decline at a similar rate, the detection of sgRNA might have some benefits in certain situations. The use of *Ct* value of SARS-CoV-2 RT-PCR of total RNA for predicting infectivity has been proposed (24, 25). However, the use of *Ct* values for interpretation of viral infectivity in patients who present for COVID-19 diagnosis before the peak viral load is reached remains a challenge, especially in those with no or mild symptoms. The interpretation of high *Ct* values (more than 30) of the RT-PCR in patients with minute symptoms during early days of infection might falsely indicate low risk of transmission, and therefore isolation precaution might not be applied. In our cohort, we found five time points in three patients at which *Ct* value further declined, and viable viruses could be isolated at the later time points (the median *Ct* values of ORF1ab and N genes before culture positivity were 32.1 and 30.4, respectively). In these patients, sgN was detected in all samples before the samples with positive culture results were obtained, while sgS was negative in two of the five time points. This suggested that sgN negativity might be used as a surrogate marker for discontinuation of isolation precaution in persons for whom the onset of symptoms is not known or uncertain.

Another challenging situation is the differentiation between reinfection and repositivity. One patient in our cohort reported a history of COVID-19. One month previously, after her COVID-19 recovery, nasopharyngeal swab had been performed, and the *Ct* values of ORF1ab and N genes were 36.1 and 36.3, respectively. At the time of recruitment, the patient reported high-risk close contact with confirmed COVID-19 patients 5 days before the symptom onset. The swab at acute respiratory infection clinic revealed RT-PCR positivity for SARS-CoV-2 at *Ct* values of ORF1ab and N genes of 33.5 and 33.0, respectively, which are in range of the values suggested for investigating suspected SARS-CoV-2 reinfection (9). It is hard to determine in this situation whether the patient had reinfection or repositivity. Because the symptoms of COVID-19 were not very specific, missed diagnosis of the true cause of symptoms might be possible in patients who had a history of COVID-19 and had RT-PCR repositivity. On the other hand, repositivity of SARS-CoV-2 RT-PCR according to the criteria in persons for whom SARS-CoV-2 was not the actual cause of the present illness would lead to unnecessary quarantine (26). In this patient, sgS and sgN were negative at the first time point after entering the study, whereas the *Ct* values of ORF1ab and N genes were 35.6 and 33.7, respectively. In this case, sgRNAs represented the more reliable marker of the virus infectivity compared to total RNAs.

In this cohort, analysis of factors associated with the duration of viral shedding showed that receipt of 2-dose vaccination was the protective factor for longer viral RNA shedding. Previously identified associated factors included older age, sex, presence of comorbidity, and the receipt of steroid (27–30). We could identify some of these factors in the univariate model. Our study identified the receipt of steroid as the only independent factor for longer isolation of viable virus and sgRNA shedding.

We accepted the limitations of this study that the cohort had a small sample size and most patients had mild symptoms. However, we were able to prospectively collect nasopharyngeal swab sampling every other day until RT-PCR negativity, and the cultures were performed on every specimen.

In conclusion, although SARS-CoV-2 sgRNAs in nasopharyngeal swab samples can be detected after the period of isolation of viable virus, sgRNAs declined faster than total RNAs. The detection of sgRNA could be used as a surrogate marker of viral infectivity. Further study on other settings, such as immunocompromised patients as well as repositivity and reinfection, should be pursued.

## MATERIALS AND METHODS

### Participants and specimen collection.

A prospective cohort of COVID-19 patients was conducted at Ramathibodi Hospital in Bangkok and Chakri Naruebodindra Medical Institute in Samut Prakan, Thailand between April and October 2021. During the study period, all COVID-19 cases in Thailand were mandatorily hospitalized for 10 to 14 days after symptom onset. Patients greater than or equal to 18 years of age who were diagnosed with COVID-19 by positive reverse transcription PCR (RT-PCR) from nasopharyngeal swab were invited to join the study. Patients who could visit the hospitals for specimen collection until RT-PCR turned negative were recruited to the study.

Nasopharyngeal swab samples were collected every other day from enrolled patients until RT-PCR negativity (cycle threshold [*Ct*] values more than 40 of both genes). The samples were collected in 2 mL of viral transport medium (VTM).

Chest radiography was performed on entry to care and at 1 week of symptom onset or if clinically indicated. Pneumonia was defined as a compatible chest film with Rama Co-RADS categories 4 or 5 (31). Treatment of the patients was provided according to a guideline issued by Department of Medical Services, Ministry of Public Health (Thailand) (32).

### Viral culture.

All collected nasopharyngeal swabs were cultured on the day of specimen collection. VeroE6 cell line expressing the transmembrane serine protease TMPRSS2 (VeroE6/TMPRSS2 cells) was maintained at 37°C and 5% CO_2_ in a humidified incubator. Cells were incubated in Dulbecco’s modified Eagle’s medium (DMEM; Gibco, NY, USA) supplemented with 10% fetal bovine serum (Gibco, NY, USA), 100 U/mL of penicillin, and 0.1 mg/mL of streptomycin. Nasopharyngeal swab contents were seeded on Vero cells and monitored for cytopathic effect (CPE) daily for 7 days, and the CPE of the virus on the cells was scored every day. The culture supernatant of the CPE-positive wells was pooled and stored at −80°C in 200-μL aliquots. If the infected cells did not show CPE by 7 days postinfection, culture supernatant of the undiluted well was used to infect new VeroE6/TMPRSS2 cells using the same infection protocol. Similar to the first round of infection, cells were observed every day for 7 days, and if cells showed CPE, the culture supernatant was collected.

Enzyme-linked immunosorbent assay (ELISA) was performed on both the initial 96-well plates of the samples that showed CPE and the reinfected 96-well plates of the samples that did not show any CPE. First, the cells were washed with 200 μL of 1× phosphate-buffered saline (PBS)/well and fixed using 200 μL/well of fresh 1:1 mixture of ice-cold methanol and acetone for 20 min on ice. Fixed cells were washed with 200 μL of 1× PBS/well and, subsequently, blocked for 1 h at room temperature with 1× PBS containing 2% bovine serum albumin (BSA) and 0.05% Tween 20. Next, cells were washed three times with PBS containing 0.05% Tween 20 (PBS-T), and 50 μL of SARS-CoV/SARS-CoV-2 nucleocapsid antibody, rabbit monoclonal antibody (MAb; Sino Biological, Beijing, China) diluted at 1:5,000 in PBS-T was added and incubated at 37°C in a humidified chamber for 1 h. In the same way, cells were washed three times with PBS-T, and 50 μL of goat anti-rabbit immunoglobulins/horseradish peroxidase (HRP; Agilent, Santa Clara, CA, USA) diluted at 1:2,000 in PBS-T was added and incubated at 37°C in a humidified chamber for 1 h. Next, plates were washed three times with PBS-T, 50 μL of KPL SureBlue TMB microwell peroxidase substrate (SeraCare, Milford, MA, USA) was added to each well, and the plates were incubated at room temperature for 10 min. Immediately after that, 50 μL of the stop solution (1 M hydrogen chloride) was added and absorbance was read using a spectrophotometer at 450/620 nm.

All the culture experiments were performed at a certified biosafety level 3, Department of Microbiology, Faculty of Science, Mahidol University.

### RNA extraction and RT-PCR.

RNA extraction was conducted at the virology laboratory at Ramathibodi Hospital on the day of specimen collection. Viral RNA was extracted from 200 μL of the samples using magLEAD (Precision System Science, Chiba, Japan) according to the manufacturer’s instructions. RT-PCR targeting SARS-CoV-2 ORF1ab and N genes (DaAn Gene, Guangdong, China) was performed immediately after RNA extraction. RT-PCR negativity was defined as *Ct* values higher than 40 in both ORF1ab and N genes (33).

### SARS-CoV-2 RT-PCR assays of sgRNAs.

Extracted RNA was stored at –80°C and was transported to the research laboratory at Faculty of Medicine Siriraj Hospital in cold chain. RT-PCR assays targeting sgS and sgN were performed on Rotor-Gene Q R09151 (Qiagen, Hilden, Germany) using the SuperScript III Platinum one-step qRT-PCR kit (Invitrogen, Waltham, MA, USA). Briefly, 50 μL reaction mixture containing 5 μL of extracted RNA, 1 μL of SuperScript III RT/Platinum *Taq* mix, 25 μL of 2× reaction mix, 1 μL ROX reference dye, 1 μL of 1 μM concentrations of both the forward and reverse primers, 1 μL of 0.5 μM probe (Sub_F, TRSB-S_R, and TRSB-S_probe [FAM] for sgS amplification and Sub_F, TRSB-N_R, and TRSB-N_probe [HEX] for sgN amplification [Table S1]), and 15 μL diethyl pyrocarbonate (DEPC)-treated water. Reactions were cycled 45 times. Each cycle consisted of reverse transcription at 55°C for 10 min, *Taq* activation at 95°C for 2 min, denaturation at 95°C for 10 sec, annealing (Fam-HEX) at 55°C for 20 sec, and extension at 60°C for 40 sec.

### Genotyping of SARS-CoV-2.

Nucleic acid was extracted from 200 μL of culture supernatant from the culture, which showed CPE or VTM of the samples that did not show CPE using the GenTiTM32 automatic extraction system (GeneAll Biotechnology, Seoul, South Korea) according to the manufacturer’s instructions, followed by Sanger sequencing of a part of the SARS-CoV-2 spike protein (34). Vector NTI Contig Express software V1.5.1 was used to generate viral sequences from raw sequencing data, and Pangolin v3.1.14 was used for assigning SARS-CoV-2 genome sequences to global lineages.

### Statistical analysis.

Descriptive statistics were presented as a number (%) for categorical variables and median (IQR) for continuous variables. *Ct* values of the gene that was not detected within 45 cycles of the RT-PCR were entered “45” for the regression analysis. A Cox regression was performed to explain the relationship between predictive factors and time to viral culture and RT-PCR negativity as follows. First, a simple Cox regression was performed by fitting each predictive factor to time-predictive variables of which *P* values of <0.20 and hazard ratio (HR) of more than 1.5 (or <0.67 for prevention factors) were simultaneously considered in the multivariate Cox model. A likelihood ratio test was used to select significant predictive factors to remain in the final model. HR and 95% confidence interval (CI) were then estimated. All statistical analyses were performed with Stata statistical software version 17.0 (Stata, College Station, TX, USA). A *P* value less than 0.05 was considered statistically significant.

### Ethics statement.

The study protocol was reviewed and approved by the Ethical Clearance Committee on Human Rights Related to Research Involving Human Subjects, Faculty of Medicine Ramathibodi Hospital, Mahidol University (MURA2021/300) and the Siriraj Institutional Review Board (Si 354/2021).
