# Supplementary material for: Detectable Duration of Viable SARS-CoV-2, Total and Subgenomic SARS-CoV-2 RNA in Noncritically Ill COVID-19 Patients: a Prospective Cohort Study
Source: Microbiol Spectr. 2022 May 23;10(3):e00503-22. doi: 10.1128/spectrum.00503-22 (PMC9241878; doi:10.1128/spectrum.00503-22)
Supplement: SUPPLEMENTAL FILE 1 — Supplemental material. Download spectrum.00503-22-s001.pdf, PDF file, 0.5 MB [file spectrum.00503-22-s001.pdf]

Supplementary figure

A

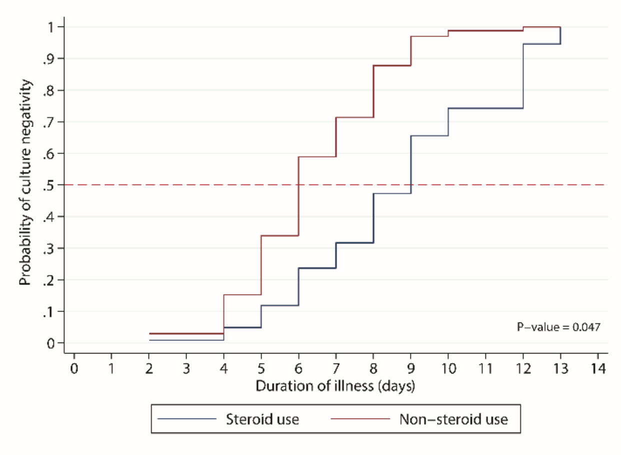

B

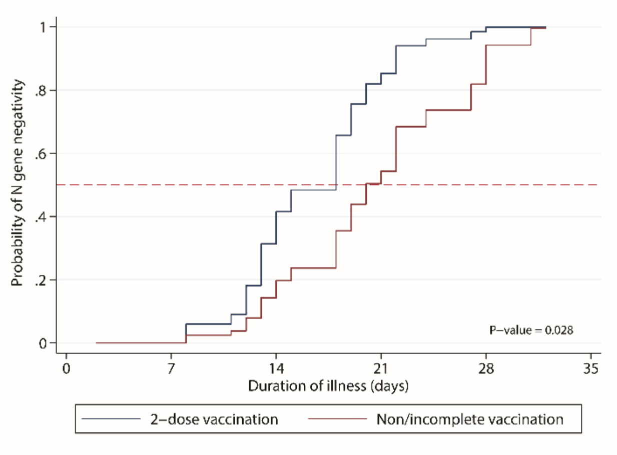

C

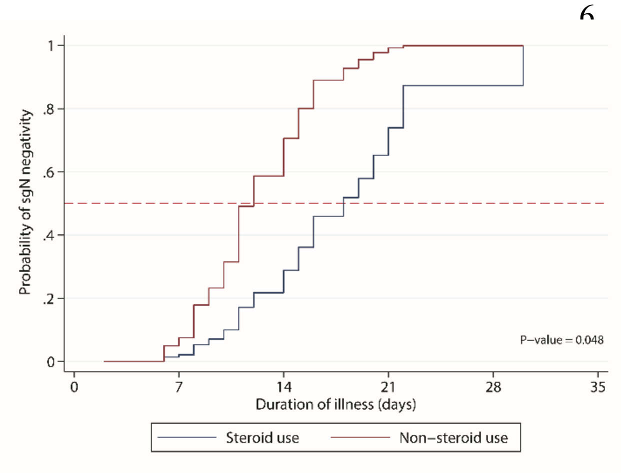

Supplementary Figure 1

Time to the negativity of SARS-CoV-2 shedding; time to viral culture negativity according to steroid use (A); time to N gene negativity according to receipt of 2-dose vaccination (B); and time to N gene subgenomic RNA negativity according to steroid use (C).

Supplementary Tables

Table 1. Primer-probe sequences designed for the amplification of SARS-CoV-2 sgRNAs.

**Primer name**	**Sequences 5'-3'**
Sub_F	CAAACCAACCAACTTTCGATCTCTTGTA
TRSB-S_R	TACCATTGGTCCCAGAGACATG
TRSB-S_probe (FAM)	CCCTGACAAAGTTTTCAGATCC
TRSB-N_R	AATTGGAACGCCTTGTCCTCGAG
TRSB-N_probe (HEX)	GCTCTCACTCAACATGGCAA

Table 2. Details of patient characteristics and results of SARS-CoV-2 detection and virus strains

Patient	Vaccine	Pneumonia	Favipiravir	Steroid	Oxygen	Strain	Detection methods	Day of illness																																		
								0	1	2	3	4	5	6	7	8	9	10	11	12	13	14	15	16	17	18	19	20	21	22	23	24	25	26	27	28	29	30	31	32	33	34
1	N	Y	Y	N	N	D614G (WT)	Virus culture					-		-		-		+		-		-		-		-		-		-												
							sgS RT-PCR					+		-		+		+		+		-		-		-		+		-												
							sgN RT-PCR					+		+		+		+		+		+		+		-		+		-												
							RT-PCR					+		+		+		+		+		+		+		+		+		-												
2	N	N	N	N	N	D614G (WT)	Virus culture			-		+		+		-		-		-		-		-		-		-														
							sgS RT-PCR			+		+		+		-		-		+		-		-		-		-														
							sgN RT-PCR			+		+		+		-		-		+		-		-		-		-														
							RT-PCR			+		+		+		+		+		+		+		+		+		-														
3	N	Y	Y	N	N	D614G (WT)	Virus culture					+		+		-		-		-		-																				
							sgS RT-PCR					+		+		+		-		-		-																				
							sgN RT-PCR					+		+		+		-		-		-																				
							RT-PCR					+		+		+		+		+		-																				
4	N	N	Y	N	N	D614G (WT)	Virus culture							-		-		-		-		N/A																				
							sgS RT-PCR							+		+		+		+		N/A																				
							sgN RT-PCR							+		+		+		+		N/A																				
							RT-PCR							+		+		+		+		N/A																				
5	N	Y	Y	N	N	Alpha (B.1.1.7)	Virus culture						-		-		-		-		-		-																			
							sgS RT-PCR						-		-		-		+		-		-																			
							sgN RT-PCR						-		-		-		-		+		-																			

							RT-PCR						+		+		+		+		+		-																			
6	N	Y	Y	Y	Y	Alpha (B.1.1.7)	Virus culture										+		+		-		-		-		-		-		-		-		-		-		-			
							sgS RT-PCR										+		+		+		+		+		+		+		-		+		-		-		-			
							sgN RT-PCR										+		+		+		+		+		+		-		-		+		+		-		-			
							RT-PCR										+		+		+		+		+		+		+		+		+		+		I		-			
7	N	Y	Y	Y	Y	Alpha (B.1.1.7)	Virus culture							+		+		N/A		-		-		-		-		-		-		-		-		-		-		-		N/A
							sgS RT-PCR							+		+		N/A		+		+		+		+		+		+		+		+		+		-		-		N/A
							sgN RT-PCR							+		+		N/A		+		+		+		+		+		+		+		+		+		-		+		N/A
							RT-PCR							+		+		N/A		+		+		+		+		+		+		+		+		+		I		+		N/A
8	N	Y	Y	N	N	Alpha (B.1.1.7)	Virus culture						-		-		-		-		-																					
							sgS RT-PCR						+		+		+		-		-																					
							sgN RT-PCR						+		+		+		-		-																					
							RT-PCR						+		+		+		I		-																					
9	Y	N	N	N	N	Alpha (B.1.1.7)	Virus culture								+		-		-		-		-		-		-		-		-		-		-							
							sgS RT-PCR								+		+		+		-		-		-		-		-		-		-		-							
							sgN RT-PCR								+		+		+		+		-		+		-		-		-		-		-							
							RT-PCR								+		+		+		+		+		+		+		+		I		I		-							
10	Y	N	Y	N	N	Alpha (B.1.1.7)	Virus culture			+		+		-		-		-		-																						
							sgS RT-PCR			+		+		+		-		+		-																						
							sgN RT-PCR			+		+		+		+		+		-																						
							RT-PCR			+		+		+		+		+		-																						
11	Y	Y	Y	Y	N	Delta (B.1.617.2)	Virus culture								+		-		-		-		-		-		N/A															
							sgS RT-PCR								+		+		-		-		+		-		N/A															

							sgN RT-PCR								+		+		+		+		+		+		N/A															
							RT-PCR								+		+		+		+		+		+		N/A															
12	N	N	N	N	N	Delta (B.1.617.2)	Virus culture			+		+		-		-		-		-																						
							sgS RT-PCR			+		+		+		-		-		-																						
							sgN RT-PCR			+		+		+		-		-		-																						
							RT-PCR			+		+		+		I		I		-																						
13	N	N	N	N	N	Alpha (B.1.1.7)	Virus culture							-		-																										
							sgS RT-PCR							-		-																										
							sgN RT-PCR							-		-																										
							RT-PCR							+		-																										
14	N	N	N	N	N	Alpha (B.1.1.7)	Virus culture													-		-		-		-		-		-		-		-		-						
							sgS RT-PCR													-		+		-		-		-		-		-		-		-						
							sgN RT-PCR													+		+		+		+		-		+		-		-		-						
							RT-PCR													+		+		+		+		+		+		+		I		-						
15	N	Y	Y	N	N	Alpha (B.1.1.7)	Virus culture					+		-		-		-		-		-		-		-		-		-												
							sgS RT-PCR					+		+		-		-		+		-		-		-		-		-												
							sgN RT-PCR					+		+		-		+		+		+		-		-		-		-												
							RT-PCR					+		+		+		+		+		+		+		+		+		-												
16	N	N	Y	Y	N	Alpha (B.1.1.7)	Virus culture				+		+		-		-		-		-		-		-		-															
							sgS RT-PCR				+		+		+		+		+		+		+		+		-															
							sgN RT-PCR				+		+		+		+		+		+		+		-		-															
							RT-PCR				+		+		+		+		+		+		+		+		-															
17	Y	N	N	N	N	Alpha (B.1.1.7)	Virus culture									-		+		+		-		-		-		-														
							sgS RT-PCR									-		+		+		+		-		-		-														
							sgN RT-PCR									+		+		+		+		-		-		-														
							RT-PCR									+		+		+		+		+		+		-														

18	N	N	N	N	N	Alpha (B.1.1.7)	Virus culture							-		-		-		-		-		-		-																
							sgS RT-PCR							+		+		+		-		-		-		-																
							sgN RT-PCR							+		+		+		+		-		+		-																
							RT-PCR							+		+		+		I		+		+		-																
19	N	N	N	N	N	Alpha (B.1.1.7)	Virus culture									-		-		-		-		-		-		-		-												
							sgS RT-PCR									+		+		+		-		-		-		-		-												
							sgN RT-PCR									+		+		+		-		-		+		-		-												
							RT-PCR									+		+		+		+		+		+		I		-												
20	N	N	N	N	N	Delta (B.1.617.2)	Virus culture								-		-		-		-		-																			
							sgS RT-PCR								+		+		-		-		-																			
							sgN RT-PCR								+		+		-		-		-																			
							RT-PCR								+		+		+		I		-																			
21	Y	N	Y	N	N	Delta (B.1.617.2)	Virus culture			-		-		-		-																										
							sgS RT-PCR			+		+		+		-																										
							sgN RT-PCR			+		+		+		-																										
							RT-PCR			+		+		+		-																										
22	Y	N	N	N	N	Delta (B.1.617.2)	Virus culture						+		-		-		-		-																					
							sgS RT-PCR						+		+		+		-		-																					
							sgN RT-PCR						+		+		+		-		-																					
							RT-PCR						+		+		+		I		-																					
23	Y	Y	Y	Y	Y	Delta (B.1.617.2)	Virus culture					+		+		+		-		-		-		-		-																
							sgS RT-PCR					+		+		+		+		+		+		+		-																
							sgN RT-PCR					+		+		+		+		+		+		+		-																
							RT-PCR					+		+		+		+		+		+		+		-																
24	N	Y	Y	N	N	Delta (B.1.617.2)	Virus culture					+		+		-		-		-		-		-		-																
							sgS RT-PCR					+		+		+		-		-		-		-		-																
							sgN RT-PCR					+		+		+		+		-		-		-		-																

							RT-PCR					+		+		+		+		+		+		+		-																
25	Y	N	N	N	N	N/A	Virus culture											-		-		-																				
							sgS RT-PCR											-		-		-																				
							sgN RT-PCR											-		-		-																				
							RT-PCR											+		+		-																				
26	N	N	N	N	N	Delta (B.1.617.2)	Virus culture						+		-		-		-																							
							sgS RT-PCR						+		+		+		-																							
							sgN RT-PCR						+		+		+		-																							
							RT-PCR						+		+		+		-																							
27	Y	N	Y	N	N	Delta (B.1.617.2)	Virus culture							-		-		-		-		-		-		-																
							sgS RT-PCR							+		+		+		+		+		-		-																
							sgN RT-PCR							+		+		+		+		+		-		-																
							RT-PCR							+		+		+		+		+		I		-																
28	Y	N	N	N	N	Delta (B.1.617.2)	Virus culture								-		-		-		-																					
							sgS RT-PCR								+		+		-		-																					
							sgN RT-PCR								+		+		-		-																					
							RT-PCR								+		+		+		-																					
29	Y	N	Y	N	N	Delta (B.1.617.2)	Virus culture					-		-		-		-		-		-																				
							sgS RT-PCR					+		+		+		-		+		-																				
							sgN RT-PCR					+		+		+		+		-		-																				
							RT-PCR					+		+		+		I		I		-																				
30	N	N	N	N	N	Delta (B.1.617.2)	Virus culture					+		+		-		-		-		-		-		-		-		-		-										
							sgS RT-PCR					+		+		+		+		+		-		-		-		+		-		-										
							sgN RT-PCR					+		+		+		+		+		-		-		-		-		-		-										
							RT-PCR					+		+		+		+		+		+		+		+		+		I		-										
31	Y	N	N	N	N	Delta (B.1.617.2)	Virus culture					+		+		-		-		-		-																				
							sgS RT-PCR					+		+		+		-		+		-																				

							sgN RT-PCR					+		+		+		-		-		-																				
							RT-PCR					+		+		+		+		+		-																				
32	N	N	N	N	N	Delta (B.1.617.2)	Virus culture				+		+		-		-		-		-		-		-		-															
							sgS RT-PCR				+		+		+		-		-		-		+		+		-															
							sgN RT-PCR				+		+		+		-		-		-		+		-		-															
							RT-PCR				+		+		+		+		+		+		+		I		-															
33	N	N	Y	N	N	Delta (B.1.617.2)	Virus culture				+		-		-		-		-		-																					
							sgS RT-PCR				+		+		+		+		-		-																					
							sgN RT-PCR				+		+		+		-		+		-																					
							RT-PCR				+		+		+		+		+		-																					
34	N	N	N	N	N	Delta (B.1.617.2)	Virus culture			+		-		-		-		-		-		-		-		-																
							sgS RT-PCR			+		+		+		-		-		-		-		-		-																
							sgN RT-PCR			+		+		-		-		-		-		-		-		-																
							RT-PCR			+		+		+		+		I		I		I		+		-																
35	Y	N	Y	N	N	Delta (B.1.617.2)	Virus culture				+		-		+		-		-		-		-		-		-		-													
							sgS RT-PCR				+		+		+		+		+		+		-		+		-		-													
							sgN RT-PCR				+		+		+		+		+		+		-		-		-		-													
							RT-PCR				+		+		+		+		+		+		+		+		+		-													
36	Y	N	Y	N	N	Delta (B.1.617.2)	Virus culture						-		-		-		-		-		-		-		-															
							sgS RT-PCR						+		+		+		-		-		-		-		-															
							sgN RT-PCR						+		+		+		-		-		-		-		-															
							RT-PCR						+		+		+		+		+		I		I		-															

Abbreviations: +, positive; -, negative; I, inconclusive; N/A, not available; N, no; Y, yes.
